# The ‘Antiretrovirals, Sexual Transmission Risk and Attitudes’ (ASTRA) Study. Design, Methods and Participant Characteristics

**DOI:** 10.1371/journal.pone.0077230

**Published:** 2013-10-15

**Authors:** Andrew Speakman, Alison Rodger, Andrew N. Phillips, Richard Gilson, Margaret Johnson, Martin Fisher, Ed Wilkins, Jane Anderson, Rebecca O’Connell, Monica Lascar, Kazeem Aderogba, Simon Edwards, Jeffrey McDonnell, Nicky Perry, Lorraine Sherr, Simon Collins, Graham Hart, Anne M. Johnson, Alec Miners, Jonathan Elford, Anna-Maria Geretti, William J. Burman, Fiona C. Lampe

**Affiliations:** 1 Research Department of Infection & Population Health, UCL, London, United Kingdom; 2 The Royal Free Centre for HIV Medicine, Ian Charleson Day Centre, Royal Free Hospital, London, United Kingdom; 3 Brighton and Sussex University Hospitals NHS Trust, Brighton, United Kingdom; 4 Pennine Acute Hospitals NHS Trust, Manchester, United Kingdom; 5 Homerton University Hospital, London, United Kingdom; 6 Barts Health NHS Trust, London, United Kingdom; 7 Eastbourne Sexual Health Centre, Eastbourne, United Kingdom; 8 Mortimer Market Centre, Central and North West London Community Foundation Trust, London, United Kingdom; 9 HIV i-Base, London, United Kingdom; 10 London School of Hygiene and Tropical Medicine, London, United Kingdom; 11 City University London, London, United Kingdom; 12 Institute of Infection and Global Health, University of Liverpool, Liverpool, United Kingdom; 13 Denver Public Health, Denver, Colorado, United States of America; Instituto de Pesquisa Clínica Evandro Chagas/Fundação Oswaldo Cruz, Brazil

## Abstract

Life expectancy for people diagnosed with HIV has improved dramatically however the number of new infections in the UK remains high. Understanding patterns of sexual behaviour among people living with diagnosed HIV, and the factors associated with having condom-less sex, is important for informing HIV prevention strategies and clinical care. In addition, in view of the current interest in a policy of early antiretroviral treatment (ART) for all people diagnosed with HIV in the UK, it is of particular importance to assess whether ART use is associated with increased levels of condom-less sex. In this context the ASTRA study was designed to investigate current sexual activity, and attitudes to HIV transmission risk, in a large unselected sample of HIV-infected patients under care in the UK. The study also gathered background information on demographic, socio-economic, lifestyle and disease-related characteristics, and physical and psychological symptoms, in order to identify other key factors impacting on HIV patients and the behaviours which underpin transmission. In this paper we describe the study rationale, design, methods, response rate and the demographic characteristics of the participants. People diagnosed with HIV infection attending 8 UK HIV out-patient clinics in 2011-2012 were invited to participate in the study. Those who agreed to participate completed a confidential, self-administered pen-and-paper questionnaire, and their latest CD4 count and viral load test results were recorded. During the study period, 5112 eligible patients were invited to take part in the study and 3258 completed questionnaires were obtained, representing a response rate of 64% of eligible patients. The study includes 2248 men who have sex with men (MSM), 373 heterosexual men and 637 women. Future results from ASTRA will be a key resource for understanding HIV transmission within the UK, targeting prevention efforts, and informing clinical care of individuals living with HIV.

## Introduction

Effective anti-retroviral therapy (ART) has resulted in a dramatic improvement in life expectancy for those infected with Human Immunodeficiency Virus (HIV) [1] [2] [3], at the same time as an increase in HIV prevalence in the United Kingdom (UK). About 96,000 people were estimated to be living with HIV in the UK in 2011 of whom 24% are undiagnosed [4]. Of the 96,000 it is estimated that 40,100 (42%) are men who acquired HIV from sex with men, 51,500 (54%) are men and women who acquired HIV through heterosexual sex and 2,300 (3%) acquired HIV through sharing drug injection equipment. The majority of those acquiring HIV through heterosexual sex (around 60%) are of African origin. Of those who are aware of their HIV infection and are under care, about 84% are taking ART. Despite continued prevention efforts, the numbers of new HIV infections detected in the UK continues to rise among men who have sex with men (MSM), with over 3000 new diagnoses in 2011 [4]. Many individuals infected with HIV by heterosexual transmission were born outside the UK and acquired HIV in their country of origin, however there is evidence of ongoing heterosexual transmission within the UK [5] [6].

HIV incidence in the UK is primarily driven by patterns of sexual risk behaviour among people with and without HIV infection. The key risk behaviour is referred to here as the amount of ‘condom-less sex’ within the population, but at an individual level transmission risk is also dependent on numbers and types of sexual partner as well as the number of vaginal or anal sex acts without a condom. Community-based studies have reported that, from the mid-1990s until recently, rates of condom-less anal intercourse have increased among MSM [7] [8] [9] [10] [11]. These trends occurred co-incident with the advent of effective ART, raising debate as to whether a new optimism surrounding HIV treatment and prognosis was one of the driving factors in changes in risk behaviour [12] [13].

Among HIV-infected individuals, a significant proportion of MSM (10-30%), and a lower proportion of heterosexual individuals, report recent condom-less sex with partners of HIV-negative status or unknown HIV status [11] [14] [15] [16] [17] [18] [19] [20], thereby posing a risk of HIV transmission to an uninfected person. An important question is whether use of ART tends to lead to increases in condom-less sex at an individual level. Early evidence gave little suggestion of this phenomenon. A meta-analysis based on studies from 1996 to 2003 showed that there was considerable heterogeneity in study results concerning the association of ART use with sexual risk behaviour (with several statistically significant results in both directions), but overall found no evidence of association when combining these differing estimates [21].

Subsequent studies based in HIV clinics (carried out up to 2005) found either no association between ART use (and/or viral suppression) and sexual risk behaviour [15] [18], or have found that patients on ART in fact tended to have somewhat lower levels of sexual risk behaviour than patients not taking ART [22] [23]. Similarly, in the SMART trial of treatment interruption, the ART continuation treatment strategy was associated with lower rates of sexual risk behaviour than the ART interruption treatment strategy [24]. 

However, over recent years there has been a fundamental change in the messages and information received by HIV-infected individuals regarding transmission risk. There has been increasing awareness that successful virological suppression with ART may lead to a profound reduction in an individual’s infectiousness. 

In 2008, an internationally well respected group of Swiss HIV clinicians and researchers issued a statement indicating that the risk of transmitting HIV through unprotected sex is extremely low if the infected partner has a suppressed plasma viral load and no other sexually transmitted disease [25]. The “Swiss Statement” received widespread attention in the UK and world-wide, and caused huge controversy and debate [26]. Recent information from The Swiss HIV Cohort Study suggests that sexual behaviour patterns may be changing among HIV diagnosed individuals in Switzerland, with participants in stable partnerships on ART reporting higher levels of condom-less sex since the publication of the “Swiss Statement.” [27]. 

However, the effect of these changing messages about ‘safe sex’ on the sexual lifestyles of HIV-diagnosed individuals in the UK is not known. The interplay between ART use, perceived viral load status, and sexual behaviour may be complex: few existing studies have assessed the role of potentially important co-factors [21], such as time since diagnosis, time since starting ART, ART side-effects, health and well-being, psychological distress, HIV disclosure, and specific beliefs regarding viral suppression and infectiousness. In addition, the extent to which HIV-diagnosed individuals have accurate knowledge of their latest viral load is unclear.

The evidence that reductions in HIV viral load are associated with a decrease in infectivity comes from longitudinal studies [28], population studies [29], and more recently from a randomised trial. In the HPTN 052 trial, it was found that successful reduction of viral load via early ART resulted in a significantly reduced risk of HIV transmission between heterosexual individuals in stable partnerships [30]. There is therefore increasing interest in a policy of ART for all individuals who are diagnosed with HIV in the UK (rather than delaying ART until an individual’s CD4 count falls below 350/mm^3^ as is recommended in current British HIV Association guidelines)[31] [32]. A policy of early ART for all people diagnosed with HIV would have the potential to reduce HIV transmission via reduction in infectiousness of HIV-diagnosed individuals. However, the individual clinical benefit of early versus delayed ART is uncertain, and is currently being evaluated in an international randomised trial which is expected to finish in 2016 (the START Study – http://www.thestartstudy.org).

Understanding the *current* relationship between ART use, viral suppression and sexual behaviour among HIV-diagnosed individuals is particularly important. If ART use were to be associated with significant changes in sexual behaviour, in particular a substantial increase in levels of condom-less sex, this could potentially offset or lessen the impact of a policy of early ART on HIV transmission.

In this context the ASTRA study was designed to investigate current patterns of sexual behaviour in a large unselected sample of HIV-infected patients under care in the UK, and among key demographic subgroups (MSM; Black African men and women; recently diagnosed people), and to assess the associations of ART use, perceived viral suppression, and other factors with condom-less vaginal or anal sex. The study is intended to help improve understanding of the current link between ART use and sexual behaviour which is particularly important in assessing the potential public health impact of a strategy of early ART. The study also directly investigated attitudes to starting ART among HIV patients not currently treated, and will therefore provide information on the acceptability of a strategy of widespread treatment and on attitudes to starting ART early to reduce infectiousness, if there is no clinical benefit to individual health.

The detailed objectives of the ASTRA study are as follows:

(i) to assess patterns of sexual behaviour among HIV-diagnosed patients under care in the UK, and to estimate levels of recent condom-less vaginal or anal sex with partners of unknown or negative HIV status, according to key demographic subgroups (MSM, Black African men and women, recently diagnosed people)
(ii) to assess whether ART use, self-reported current undetectable viral load, and other factors (demographic/social factors, psychological and physical symptoms, quality of life, lifestyle factors, HIV-related factors, time since starting ART and ART adherence) are associated with condom-less sex with HIV-negative or unknown status partners
(iii) to investigate patients’ beliefs regarding the effect of ART, and undetectable viral load, on HIV transmission risk (transmission risk beliefs), and the association of transmission risk beliefs with sexual behaviour
(iv) to assess the association between self-reported and clinically-documented viral load status
(v) to investigate attitudes to starting immediate ART, among ART naïve patients and to assess whether transmission risk beliefs and demographic, health, lifestyle and HIV-related factors are associated with these attitudes
(vi) to assess the prevalence of, and interrelationships between questionnaire-assessed health and lifestyle factors among HIV-diagnosed patients
(vii) in a subset of patients, to investigate the association of questionnaire-assessed factors with laboratory and clinical outcomes (including virological failure, and major morbidity and mortality).

The current paper sets out the key aspects underlying the ASTRA study, including its rationale, design, methods, response rate and the demographic characteristics of the participants. Subsequent publications will address detailed research questions based on the data collected from the participants.

## Design and Methods

### Population and setting

A cross-sectional self-administered questionnaire study was conducted at eight out-patient HIV clinics in the UK. The study aimed to include a sample of all the HIV out-patients attending at each centre, regardless of ART status. The exclusion criteria were as follows: those under 18; those unable to understand the questionnaire due to language or cognitive difficulties; and those too ill or distressed to complete the questionnaire. Additionally the recruitment period was of sufficient duration (~12 months) so that infrequent clinic attenders were likely to be included in those invited to participate.

The eight clinical centres were selected based on previously successful research collaborations and on the understanding that they could provide access to significant numbers of HIV diagnosed patients, including the key demographic subgroups in the UK (MSM and Black Africans). The centres were as follows:

• Royal Sussex County Hospital, Brighton
• Eastbourne Sexual Health Clinic
• Homerton University Hospital, London
• Mortimer Market Clinic, London
• Newham University Hospital, London
• North Manchester General Hospital
• Royal Free Hospital, London
• Whipps Cross University Hospital, London

Seven of the eight clinics have a patient population size greater than the national average of 332 (based on the 211 HIV out-patient clinics in England, Wales and Northern Ireland [33]). Further details of recruitment periods and the estimated total patient population for each clinic are shown in Table 1.

**Table 1:** Recruitment details for the eight ASTRA study clinical centres 2011 - 2012.

	**Royal Sussex County Hospital Brighton**	**Eastbourne Sexual Health Clinic**	**Homerton University Hospital**	**Mortimer Market Clinic**	**Newham University Hospital**	**North Manchester General Hospital**	**Royal Free Hospital**	**Whipps Cross University Hospital**	**TOTAL**
Location	Brighton	Eastbourne	East London	Central London	East London	Manchester	North London	East London	-
Date recruitment began	14/02/2011	19/10/2011	23/02/2011	07/04/2011	16/07/2012	22/02/2011	02/02/2011	19/07/2012	-
Date recruitment ended	19/12/2011	14/02/2012	23/12/2011	02/12/2011	21/12/2012	17/02/2012	16/12/2011	20/12/2012	-
Length of study period in days	309	119	304	240	159	361	318	155	-
Estimate of patients seen in all clinical sessions during recruitment period	1956	122	844	3740	819	1678	2984	423	12566
Eligible patients approached	787	104	465	1317	276	732	1336	95	5112
Patients consenting (as % of approached = consent rate)	718 (91%)	82 (79%)	374 (80%)	1132 (86%)	196 (71%)	523 (71%)	1087 (81%)	88 (93%)	4200 (82%)
Participants agreeing to clinical linkage (as % of those consenting)	654 (91%)	76 (93%)	326 (87%)	1035 (91%)	159 (81%)	505 (97%)	953 (88%)	84 (95%)	3792 (90%)
Patients responding = questionnaires received (as % of approached = response rate)	523 (66%)	61 (59%)	269 (58%)	907 (69%)	179 (65%)	355 (48%)	899 (67%)	65 (68%)	3258 (64%)
Respondents agreeing to clinical linkage (as % of respondents)	484 (93%)	60 (98%)	233 (87%)	842 (93%)	147 (82%)	345 (97%)	809 (90%)	63 (97%)	2983 (92%)

### Sample size

The sample size for ASTRA was calculated to detect an absolute difference of 4% in the overall proportion of participants reporting condom-less sex with an HIV-negative or unknown HIV-status partner (CLS-D), comparing those taking ART and those not taking ART. The calculations were based on 80% power and 5% 2-sided significance level and assumed that 75% of HIV out-patients are on ART and the proportion of patients off ART reporting CLS-D is 12% [15] [17] [18] [19] [20]. The required sample size based on these assumptions was 3349 participants.

### Recruitment

**Figure 1:**
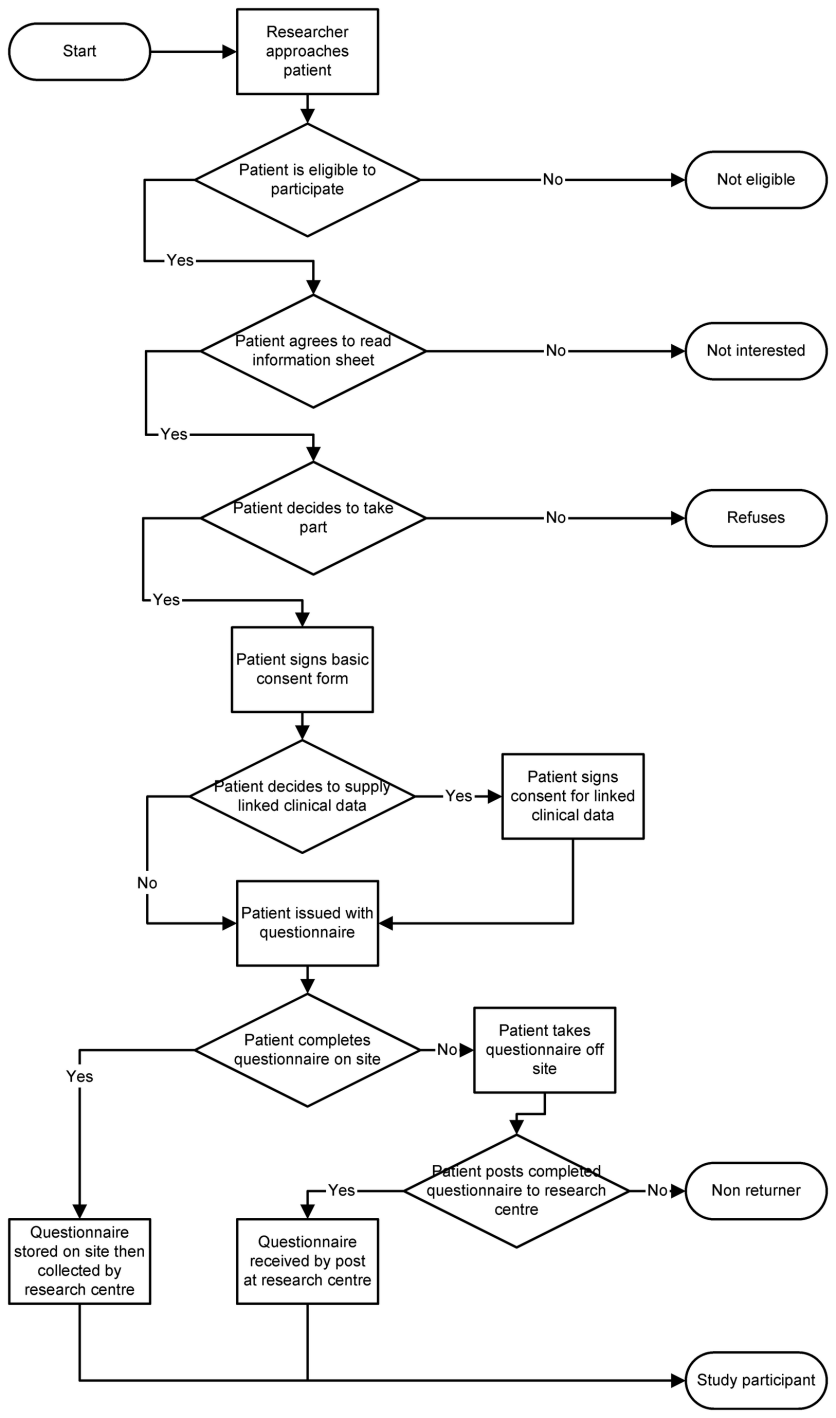
Flowchart of ASTRA clinic recruitment and questionnaire collection procedures.

Recruitment to the study took place between February 2011 and December 2012 at different periods in the 8 centres (full details in Table 1). A flowchart of recruitment procedures for the study is included (see Figure 1). 

The study clinic teams identified specific routine clinical sessions during which recruitment would take place throughout the study period. During these selected recruitment sessions, consecutive eligible patients were approached by clinic staff and invited to participate. In some clinics this process took place in the waiting area; in others it took place during routine consultations. 

Patients who expressed an interest were given a patient information sheet that contained full details of the research project, including contact details for the research team. The research staff explained the purpose of the study and its potential benefits and answered any questions or concerns (see the ‘Ethics’ section below for a summary of measures undertaken to address patient issues).

If the patient agreed to participate they were required to sign the study consent form. As part of the consent process it was explained that the latest viral load and CD4 count would be recorded for all those participating, but there was also an optional agreement to allow linkage of participants’ questionnaire data with further routine clinical information (see the ‘Linked clinical data’ section below for further details on linked clinical data).

Where possible, participants were encouraged to complete the questionnaire at the clinic (either before or after their appointment, or while waiting for tests or prescriptions). In several clinics, participants could maintain sufficient privacy in the waiting area to complete questionnaires there (although all participants were made aware that they could ask for a private area if necessary). In other clinics a separate private room away from the waiting area was routinely used for completing the questionnaire. If the participant could not spare the time to complete the questionnaire in clinic, he/she was requested to take the questionnaire away from the clinic, complete it, and return it using a provided pre-paid envelope addressed to the study management centre.

Participants were told that the questionnaire would take between 15 and 30 minutes to complete. They were also given an envelope and asked to seal the completed questionnaire in it to ensure that their answers were kept confidential from clinic staff. Completed questionnaires were collected in the clinic and transferred regularly to the study management centre.

For patients who took the questionnaire off site, there were procedures for reminding those who did not return it within a period of approximately one month. In some clinics text message reminders were sent or phone calls made to those who had not returned questionnaires. In other clinics, those attending for repeat appointments were identified and reminded personally by clinical staff.

### Data processing

Questionnaires were identified only by a unique study number and participants were instructed not to write their name or clinic number on the questionnaire to maintain their anonymity (see the ‘Ethics’ section below for a summary of measures undertaken to ensure confidentiality).

Details of all the clinic attendees approached for the study were collected in a study log maintained securely and updated daily at each clinical site. The study log contained study numbers, clinic identifiers and details of consent status for all patients invited to participate in the study. The latest viral load and CD4 count was recorded for those who agreed to participate. Clinic research staff were asked to enter the latest laboratory result values known to the participant at the time the questionnaire was issued. Contact details of participants were also entered in the log if the participant had agreed to receive reminders to return the questionnaire. Information from the study log at each clinical centre was transferred on a regular basis to the study management centre. The information transferred did not include clinic identifiers or contact details of the participants, ensuring that the version of the study log held at the study management centre was effectively anonymised.

Regular reports were sent from the study management centre to each site during the recruitment period, detailing trends and overall progress in recruitment for each of the study sites. In addition regular checks were made on the completeness and quality of the study log and its concordance with received questionnaires. A further checking process identified 72 cases where participants filled out a questionnaire more than once. In these cases only the first completed questionnaire was included in the analysis.

Questionnaires received at the management centre were digitised by an external data processing contractor. Each paper questionnaire was checked for legibility, digitally scanned and the resulting images were used as the source for two manual data entry rounds with subsequent quality checking. The completed data entry batches delivered by the contractor were checked for accuracy at the study management centre by fully examining a 5% sample (169 questionnaires). The data entry error rate using this method was found to be 0.06%.

The original anonymised study datasets, including scanned images of the questionnaires are stored at the study management centre in encrypted digital form. They are preserved by being duplicated and stored on managed servers with regular backup and professional administration. The original paper questionnaires are stored securely in locked cabinets. The study datasets will be made freely and readily available to the research community after a suitable interval in a form that ensures that participant anonymity and confidentiality is maintained.

### Study questionnaire

An initial questionnaire design was printed in A4 format and piloted at 4 study sites in December 2010, using the recruitment procedures described above. Following feedback from participants and research staff, a few minor revisions were made to the final questionnaire, the patient information sheet and consent form. These changes were submitted as amendments for ethical approval and were incorporated into the final version employed during the main recruitment period, which commenced in February 2011.

The final questionnaire consisted of a printed A5 booklet, with versions for men (28 page questionnaire) and women (24 page questionnaire). Translated French versions of both questionnaires were also available in A4 format. However uptake of this option was minimal (n=14).

The study originally envisaged that sites would employ the option of either paper questionnaires or a computer assisted self-interviewing (CASI) version on a small handheld laptop. The CASI version of the questionnaire was developed and tested as part of the study. However, initial assessments showed that there were difficulties managing the administration of both versions concurrently, particularly for a single staff member aiming to recruit significant numbers of participants each day. Patients and staff also preferred using the paper version. Therefore the paper version was used exclusively at all sites throughout the study.

The questionnaire sought detailed information on the following factors:

• **Demographic and social factors**: including gender, date of birth ethnicity, education, employment, housing, financial status, religion, sexuality, relationship status (whether in long-term partnership and HIV-status of partner), country of birth, time living in the UK (if non-UK birth) immigration status, number of children
• **HIV-related factors**: diagnosis date, number of years attended the current clinic, current CD4 count (grouped into five categories including ‘don’t know’), likely route of infection, disclosure of HIV status
• Health, symptoms and well-being:
• Physical and psychological symptoms (modified version of Memorial Symptom Assessment Scale Short-Form) [34] [35]
• Depression (PHQ-9) [36]
• Anxiety (GAD-7) [37]
• Health-related quality of life (EuroQoL 5D) [38]

Social support (modified version of the Duke–UNC Functional Social Support Questionnaire – FSSQ) [39]

• **Relevant medical history**: including recently diagnosed sexually transmitted infections (STIs), symptoms of STIs, diagnosed hepatitis C, treatment for depression, treatment for other mental health problems, pregnancy status for women
• **HIV-transmission risk**: whether transmission risk recently discussed with clinical staff, beliefs about transmission risk in relation to ART and undetectable viral load
• ART-related factors:
• *for* *those* *who* *had* *ever* *taken* *ART*: date of starting ART, reasons for starting ART, perceptions about the difficulty of taking ART compared to expectations, latest viral load (‘undetectable’, ‘detectable’ or ‘don’t know’), whether ever switched ART due to virological failure; whether currently on ART (with reasons for stopping if applicable), recent non-adherence (with reasons) for current ART users only;
• *for* *those* *who* *had* *never* *taken* *ART*: preferences about starting treatment immediately if it were shown to (i) slightly reduce risk of serious illness and (ii) reduce infectiousness to a sexual partner; whether been advised to start ART (with reasons for not starting if applicable)
• **Lifestyle factors**: cigarette smoking status, usual alcohol intake, evidence of alcohol dependency (the CAGE questionnaire) [40], recent use of recreational drugs (with details) and recent use of injecting drugs
• **Sexual activity**: sexual activity (vaginal or anal sex) during the previous 3 months was ascertained separately for (i) men having sex with women (ii) men having sex with men (iii) women having sex with men. There were questions on number of partners, type of partners (long-term or other), condom-less sex with HIV-positive and HIV-negative/unknown status partners (CLS-D). For those participants who reported CLS-D in the past 3 months, there was detailed inquiry on number and type of partners, frequency of sex, type of sex (receptive or insertive anal sex and whether ejaculation occurred), reasons for not using a condom. All participants were also asked about: use of the internet to find sexual partners; group sex; receiving money or drugs for sex; whether partners had used PrEP (pre exposure prophylaxis) or PEPSE (post exposure prophylaxis after sexual exposure to HIV) to reduce the risk of HIV transmission; attitudes to disclosure of HIV to sexual partners and negotiation of condom use; and their total number of new sexual partners in the past year.

PDF copies of the questionnaires are available for download from the study website (http://www.astra-study.org). Paper versions are also available on request.

### Linked clinical data

Basic consent to participate in the study included permission to collect the latest CD4 count and HIV plasma viral load values (with dates) known to each participant and these details were recorded in the study log. 

Participants were also asked if they would consent to linkage of questionnaire data with their routine clinical data for this study over the next few years. They were told that this was an optional consent and that they could refuse and still participate in the questionnaire part of the study. The clinical data consisted of laboratory test results (including CD4 count and viral load), HIV treatment history, and other routinely available information on HIV clinical care including Acquired Immunodeficiency Syndrome (AIDS) events, serious illnesses, hospital admissions and dates of death where appropriate.

For consenting subjects, the first linkage of questionnaire data with clinical data took place shortly after the end of study recruitment. Subsequent linkages will occur on up to four additional occasions over the next five years (to allow for prospective analyses). Clinical data are provided by the clinics for each consenting participant based on study number. Clinic identifiers are not transferred to the study management centre, ensuring that the clinical data are effectively anonymised.

The clinical data for the study are stored at the study management centre in encrypted digital form. They are preserved by being duplicated and stored on managed servers with regular backup and professional administration.

### Ethics statement

The research protocol and all versions of the study documents (information sheet, consent form and questionnaire) were approved by the North West London REC 2 research ethics committee (ref 10/H0720/70). Based on these documents, the study subsequently received permission for clinical research at all participating National Health Service sites.

A summary of the measures taken to ensure the confidentiality and security of patient information is as follows:

1. care was taken to ensure the privacy of participants during questionnaire completion

2. participants were assured that their questionnaire responses would not be seen by clinic staff or recorded in clinic notes (completed questionnaires were stored in sealed envelopes and not made available to the local clinic staff)

3. data stored at the study management centre was identified only by an anonymous study number and did not contain sufficient detail to allow personal identification of responses during the data processing stage

4. measures were undertaken to ensure data security at the study management centre

5. the information sheet included a commitment to aggregated analysis with no individual data to be revealed

A summary of the measures taken to ensure that study participants could consider the issues effectively and discuss any concerns is as follows:

1. in the patient information sheet, participants were made aware that they could withdraw at any stage; they were also offered contact with clinical staff to discuss any issues of concern

2. the questionnaire noted that participants could leave out any of the questions; it also gave details of an HIV helpline and included a comments box at the end for participants to express their opinions of the study

3. a study website was set up (http://www.astra-study.org) providing access to full contact details, progress updates, background information, all the study documents and future results

### Study management

The study was managed on a day to day basis by a core group of four staff at the research centre: the HIV Epidemiology and Biostatistics Group, Research Department of Infection and Population Health, Royal Free Campus, University College London.

An advisory group was also established at the start of the study to provide guidance and support. The advisory group consisted of representatives from University College London, the University of Liverpool, HIV i-Base, the Department of Public Health in Colorado, the London School of Hygiene and Tropical Medicine and City University London.

## Results

### Recruitment

Over the two year study period a total of 5112 eligible patients were approached and asked to participate in the study. Of those approached, 4200 (82%) gave consent to take part in the study. Of those consenting, 3792 (90%) agreed to linkage of their questionnaire responses with routine clinical data. The number of questionnaires finally collected was 3258 and thus the response rate was 64% (3258/5112) of eligible patients approached and 78% (3258/4200) of those who gave consent. 2983 of the 3258 respondents (92%) agreed to linkage of their questionnaire responses with routine clinical data.

Of the 4200 consenting participants, 942 did not return a questionnaire. However when using the limited information recorded in the study log to compare those that did (n=3258) and did not (n=942) return questionnaires, these groups were found to be similar in terms of gender (% female: 19.6% versus 21.5% for returners and non-returners respectively, χ^2^(1) = 1.8, p = 0.18), proportion with HIV viral load ≤50c/mL (76.3% versus 76.4%, χ^2^(1) = 0.008, p = 0.93) and proportion with CD4 count <350/mm^3^ (18.6% versus 21.3%, χ^2^(1) =3.3, p=0.07). 

The eight participating clinics provided estimates of the number of patients seen in all clinical sessions over the same period. A total of 12566 patients attended clinic at some point during the recruitment periods meaning that, during the selected clinical sessions, the recruiters approached 41% (5112/12566) of all clinic attendees as part of our study.

Table 1 shows the recruitment periods, patient populations and response rates for the eight clinical centres.

### Characteristics of those recruited

The mean age of the 3258 participants at the time of questionnaire completion was 45 years (SD=10, range 18-88 years) and the proportions who were <30, 30-39, 40-49, 50-59, ≥60 years were 5%, 23%, 43%, 22% and 7% respectively. 2621 (80%) participants were men and 637 (20%) were women. Of the male participants, 2248 (86%) were classified as MSM and 373 (14%) as heterosexual.

In terms of ethnic origin, 2220 (68%) participants were classified as white, 614 participants (19%) were of Black African ethnicity, 125 (4%) were of other Black ethnicity, 226 (7%) were of other ethnicity and ethnic status was missing for 73 (2%). Overall, of 3202 participants with information on ART status, 2765 (86%) reported taking ART at the time of questionnaire completion.

There were differences between the characteristics of those recruited at the eight clinical centres in terms of gender, sexual orientation and ethnic origin (see Table 2).

**Table 2:** Characteristics of ASTRA respondents at the eight study clinical centres 2011 - 2012.

	**Royal Sussex County Hospital Brighton**	**Eastbourne Sexual Health Clinic**	**Homerton University Hospital**	**Mortimer Market Clinic**	**Newham University Hospital**	**North Manchester General Hospital**	**Royal Free Hospital**	**Whipps Cross University Hospital**	**TOTAL**
Number of participants (questionnaires received)	523	61	269	907	179	355	899	65	3258
Respondents as % of total sample	16%	2%	8%	28%	5%	11%	28%	2%	100%
Mean age in years at questionnaire completion (SD)	46 (11)	50 (11)	42 (9)	44 (9)	44 (11)	46 (10)	46 (9)	44 (9)	45 (10)
Gender: Men n (%*)	480 (92%)	43 (70%)	142 (53%)	805 (89%)	85 (47%)	284 (80%)	741 (82%)	41 (63%)	2621 (80%)
Gender: Women n (%*)	43 (8%)	18 (30%)	127 (47%)	102 (11%)	94 (53%)	71 (20%)	158 (18%)	24 (37%)	637 (20%)
Sexuality: MSM n (%*)	451 (86%)	35 (57%)	73 (27%)	743 (82%)	37 (21%)	235 (66%)	651 (72%)	23 (35%)	2248 (69%)
Sexuality: Heterosexual men n (%*)	29 (6%)	8 (13%)	69 (26%)	62 (7%)	48 (27%)	49 (14%)	90 (10%)	18 (28%)	373 (11%)
Ethnicity: White n (%*)	460 (88%)	45 (74%)	64 (24%)	671 (74%)	35 (20%)	251 (71%)	669 (74%)	25 (38%)	2220 (68%)
Ethnicity: Black African n (%*)	33 (6%)	8 (13%)	143 (53%)	93 (10%)	115 (64%)	75 (21%)	125 (14%)	22 (34%)	614 (19%)
Ethnicity: Black other n (%*)	2 (<1%)	1 (2%)	40 (15%)	28 (3%)	9 (5%)	6 (2%)	31 (3%)	8 (12%)	125 (4%)
Ethnicity: Other ethnicity^$^ n (%*)	28 (5%)	7 (12%)	22 (8%)	115 (13%)	20 (11%)	23 (6%)	74 (8%)	10 (15%)	299 (9%)
Self reporting currently on ART^#^ (%*)	435 (84%)	57 (93%)	227 (85%)	696 (79%)	159 (90%)	327 (93%)	809 (92%)	55 (87%)	2765 (86%)

* Percentage of total number of participants within each clinic, and overall (for total column); ^$^Includes missing ethnicity (n=73); ^#^n=3202 with data on ART status

## Discussion

The ASTRA study recruited 3258 participants from eight UK HIV out-patient clinics during 2011/2012. Although the study recruited only during selected clinical sessions at each of the eight clinics, the total number of patients invited to participate represents a large proportion of all those attending the participating clinics over the study period (5112/12566 = 41%) and questionnaires were finally obtained from 26% (3258/12566) of this total clinic population. The numbers approached and recruited in the ASTRA study are relatively large in relation to the size of the UK HIV-diagnosed population which was estimated to be about 73400 in 2011 [4]. The patients approached account for approximately 7% of the national HIV-diagnosed population, and our sample of 3258 included approximately 4.4% of all those living with diagnosed HIV in the UK. 

To our knowledge this is the largest clinic-based questionnaire study to date of HIV-diagnosed individuals in the UK. Previous large multicentre studies of HIV out-patients in the UK include the ‘What do you need’ study (1777 responses using an opportunistic sample across 107 different UK organisations in 2001 [41]); the ‘East London Study’ (1687 participants from 6 London centres in 2004/5 [42]); and the ‘Switching Study’ (778 participants from 5 centres in London and the South East in 2005/6 [19]).

In addition to the large sample size, particular strengths of the ASTRA study include the collection of comprehensive information on recent sexual activity (over the previous 3 months), the collection of both self-reported and clinic-recorded viral load for all study participants, and the very high proportion of respondents (92%) who agreed to linkage of questionnaire data with routine clinic data. 

The initial rates for consent (82%) and for consent to supply clinical data (90%) were high in this study. However the overall response rate (questionnaires received) was 64% of eligible patients approached. This value is comparable with other surveys taking place outside the clinical context that have investigated sexual behaviour (70% - [20]; 65% - [43]), but lower than some previous HIV-clinic based pen and paper questionnaires some of which achieve values above 70% [19] [42] [44] [45]. It should be noted that the 1854 non-responding eligible patients were made up of 912 direct refusals to participate and 942 consenting participants who took a questionnaire away but did not return it (approximately 18% of eligible patients in each case). It therefore seems that a major aspect of the non-response in this study was not related to the initial patient perceptions of the study but could be attributed to factors impacting after taking the questionnaires away – possibly including the length of the questionnaire, a lack of time to complete it in the clinic, or to participants reassessing the acceptability of some of the questions outside the clinic setting. It was not possible to collect any information on those people who declined to take part in the study. However, the fact that no major differences were found within the consenting group in terms of gender, viral load status and prevalence of low CD4 count between those who did and did not return a questionnaire gives some reassurance that the final study participants were representative of all those who initially agreed to take part in terms of demographic and HIV-related factors.

In some key areas, the study population reflected the UK national HIV diagnosed population. The average age of study participants was 45 years which is consistent with UK national data, in that the majority of HIV positive diagnosed individuals fall into the 35-49 age group [4]. In addition 86% of study participants reported receiving current antiretroviral therapy during the 2011-2012 study period, which is compatible with the UK national estimate for those diagnosed with HIV who are receiving ART, which is on a rising trend from 71% in 2002 to 84% in 2011 [4].

The study population differs from the UK national HIV diagnosed population with respect to proportions in key demographic sub-groups. There were 2248 MSM participants (69%), 614 Black African participants (19%) and 637 women (20%). These proportions mean that MSM are over-represented and Black Africans and women are under-represented in this study when compared to the percentages within the UK diagnosed HIV positive population as a whole (heterosexuals born in Africa - 32%, MSM - 43%, heterosexual women - 32% [4]). Nevertheless the ASTRA study has succeeded in its aim to recruit large numbers within the population groups most affected by HIV in the UK, namely MSM and Black African men and women.

The demographic characteristics of the participants also varied greatly between the different clinics, reflecting their different locations and local populations. Nevertheless the majority of participants (2319 = 71%) were recruited in London. This is relevant because the UK HIV epidemic is concentrated in London with most new diagnoses (42% in 2011) occurring there and it is where most UK local authorities with a high prevalence of diagnosed HIV infection are concentrated [4].

In summary, the size and recruitment characteristics of this study mean that it is a substantive sample of those diagnosed with HIV in the UK. Its results will therefore have implications for understanding HIV transmission within the UK, and for targeting of national prevention efforts. The results will also give further insights into the relationships between socio-demographic factors, physical and psychological symptoms, lifestyle factors, health-related quality of life and ART outcomes among individuals living with HIV in the UK.
